# Diurnal preference, mood and the response to morning light in relation to polymorphisms in the human clock gene *PER3*

**DOI:** 10.1038/s41598-017-06769-w

**Published:** 2017-07-31

**Authors:** M. Turco, A. Biscontin, M. Corrias, L. Caccin, M. Bano, F. Chiaromanni, M. Salamanca, D. Mattei, C. Salvoro, G. Mazzotta, C. De Pittà, B. Middleton, D. J. Skene, S. Montagnese, R. Costa

**Affiliations:** 1 Department of Medicine, University of Padova, Padova, Italy; 2 Department of Biology, University of Padova, Padova, Italy; 3 Chronobiology, Faculty of Health and Medical Sciences, University of Surrey, Guildford, United Kingdom

## Abstract

*PER3* gene polymorphisms have been associated with differences in human sleep-wake phenotypes, and sensitivity to light. The aims of this study were to assess: *i*) the frequency of allelic variants at two *PER3* polymorphic sites (rs57875989 length polymorphism: *PER3* ^4^, *PER3* ^5^; rs228697 SNP: *PER3* ^C^, *PER3* ^G^) in relation to sleep-wake timing; *ii*) the effect of morning light on behavioural/circadian variables in *PER3* ^4^ */PER3* ^4^ and *PER3* ^5^ */PER3* ^5^ homozygotes. 786 Caucasian subjects living in Northern Italy donated buccal DNA and completed diurnal preference, sleep quality/timing and sleepiness/mood questionnaires. 19 ***PER3*** ^**4**^ ***/PER3*** ^**4**^ and 11 ***PER3*** ^**5**^ ***/PER3*** ^**5**^ homozygotes underwent morning light administration, whilst monitoring sleep-wake patterns and the urinary 6-sulphatoxymelatonin (aMT6s) rhythm. No significant relationship was observed between the length polymorphism and diurnal preference. By contrast, a significant association was observed between the *PER3* ^G^ variant and morningness (OR = 2.10), and between the *PER3* ^G^-*PER3* ^4^ haplotype and morningness (OR = 2.19), for which a mechanistic hypothesis is suggested. No significant differences were observed in sleep timing/aMT6s rhythms between *PER3* ^5^ */PER3* ^5^ and *PER3* ^4^ */PER3* ^4^ subjects at baseline. After light administration, *PER3* ^4^ */PER3* ^4^ subjects advanced their aMT6s acrophase (p < 0.05), and showed a trend of advanced sleep-wake timing. In conclusion, significant associations were observed between *PER3* polymorphic variants/their combinations and both diurnal preference and the response to light.

## Introduction

Clock genes and their products are associated with the current molecular model for the genesis of circadian rhythms^1^. Allelic variants of clock genes affect the period and the phase of circadian rhythms^2^.

An important component of the biological clock is the *Period* (*PER*) gene family. The heterodimers formed by PER3 protein with PER1/2 and cryptochrome 1/2 (CRY1/2) enter the nucleus, inhibit CLOCK/BMAL1-mediated transcription^3^, thus contributing to the output pathway of circadian oscillations^4^. The human *PER3* gene shows higher levels of polymorphism compared to the other PER paralogs^5^, suggesting that *PER3* may account for individual differences in human circadian and sleep phenotypes.

The coding region of the *PER3* gene contains a variable-number tandem-repeat (VNTR) polymorphism, i.e. a motif encoding 18 amino acids is repeated either four (*PER3* ^4^) or five (*PER3* ^5^) times in exon 18^6^ (rs57875989, NM_001289861.1:c.3002-13_3042del54). The repeated unit contains a cluster of putative phosphorylation sites, and the polymorphism may influence PER3 function. There is some evidence that, in humans, *PER3* may be related to diurnal preference, or the personal inclination to be more/less active at different times of the waking day. The *PER3* ^4^ allelic variant has been associated with physiological and pathological eveningness^6, 7^ while the *PER3* ^5^ allele has been associated with morningness^7^. In addition, the *PER3* ^5^ allele has been associated with enhanced suppression of nocturnal melatonin by administration of light at night^8^. However, the effect of light in the morning, which is often utilised for treatment purposes (i.e. seasonal affective disorder, delayed sleep-wake rhythms, irregular schedules etc.^9, 10^) has not been tested within this context.

Recently, a SNP (rs228697, NM_001289861.1:c.2590 C> G) in exon 17 of *PER3* that causes the amino acid substitution P864A has also been associated with diurnal preference^11^, with the 2590G allele (864 A) being more prevalent in evening types. The P864A substitution would disrupt two potential SH3-binding motif domains in PER3, most likely altering the binding properties of the protein. The scaffolding protein NCK contains SH3 binding motifs which could mediate the interactions with PER3 and with the subunit γ2 of Casein Kinase 1 (CK1)^12^. CK1δ and ε are known to regulate PER proteins stability, activity and subcellular localization, but it cannot be excluded that CK1γ may also be involved in post-translational regulation of PER proteins.

Thus the aim of this study was to assess the frequency of allelic variants at two *PER3* polymorphic sites in a large population of healthy volunteers, and their relationship to diurnal preference, sleep-wake timing and mood. In addition, the effect of an 8-day course of morning light administration on behavioural and circadian variables in a group of well-characterised *PER3* ^4^ */PER3* ^4^ and *PER3* ^5^ */PER3* ^5^ homozygotes was also studied.

## Results

### Genotype, diurnal preference and sleep-wake variables

The HÖ questionnaire was completed by 786 subjects (541 females); average HÖ score was 50.2 ± 10.4 (range: 21–78). Diurnal preference was distributed as follows: 22 extreme evening (3%), 147 moderate evening (19%), 436 intermediate (55%), 163 moderate morning (21%) and 18 extreme morning (2%). HÖ scores were in agreement with daily sleep-wake timing in the 494 subjects for whom sleep diaries were also available [i.e. wake up time (decimal h): evening types 8.5 ± 1.2 vs. intermediate types 7.7 ± 0.7 vs. morning types: 6.9 ± 0.6; ANOVA p < 0.001, each group significantly different from all others on post-hoc, with p < 0.001].

Both the VNTR genotype and HÖ questionnaire data were available for 679 individuals (459 females). Of these, 271 (39.9%) subjects were *PER3* ^4^ */PER3* ^4^, 97 (14.3%) *PER3* ^5^ */PER3* ^5^ and 311 (45.8%) *PER3* ^4^ */PER3* ^5^.

This population was shown to be in Hardy-Weinberg equilibrium (χ^2^ = 0.259, DF = 1, p = 0.610, Table 1), with no significant differences in genotype distribution between females and males (χ^2^ = 2.976, DF = 2, p = 0.226). The distribution of diurnal preference in relation to VNTR genotype is presented in Table 1. No preferential associations between genotype/allele and diurnal preference were observed (Wald test: p = 0.737). This also held true when intermediate types were excluded and when extreme morning/morning types and extreme evening/evening types were pooled (Cochran-Armitage test χ^2^ = 0.069, p = 0.793).

**Table 1:** *PER3* genotypes at rs57875989 and minor allele frequencies, by diurnal preference.

	rs57875989	Extreme Morning n (%)	Morning n (%)	Intermediate n (%)	Evening n (%)	Extreme evening n (%)	Total n (%)	MAF
All	4/4	7 (38.9)	59 (43.7)	142 (38.0)	57 (43.2)	6 (30.0)	271 (39.9)	0.3719
	4/5	6 (33.3)	54 (40.0)	183 (48.9)	56 (42.4)	12 (60)	311 (45.8)	
	5/5	5 (27.8)	22 (16.3)	49 (13.1)	19 (14.4)	2 (10)	97 (14.3)	
Females	4/4	6 (46.1)	45 (47.4)	104 (39.5)	40 (44.9)	3 (27.3)	198 (42.0)	0.3599
	4/5	4 (30.8)	34 (35.8)	126 (47.9)	37 (41.6)	6 (54.5)	207 (43.9)	
	5/5	3 (23.1)	16 (16.8)	33 (12.6)	12 (13.5)	2 (18.2)	66 (14.0)	
Males	4/4	1 (20.0)	14 (35.0)	38 (34.2)	17 (39.5)	3 (33.3)	73 (35.1)	0.3990
	4/5	2 (40.0)	20 (50.0)	57 (51.3)	19 (44.2)	6 (66.7)	104 (50.0)	
	5/5	2 (40.0)	6 (15.0)	16 (14.5)	7 (16.3)	0 (0.0)	31 (14.9)	

MAF: Minor Allele Frequency. No preferential associations between genotype/allele and diurnal preference were observed (Wald test: p = 0.737). This held true also when intermediate types were excluded and extreme morning/morning types and extreme evening/evening types were pooled (Cochran-Armitage test χ^2^ = 0.069, p = 0.793).

Subjects with available VNTR genotype (and available, residual DNA) plus diurnal preference data were selected for SNP rs228697 genotype determination, based on their diurnal preference score. Morning/extreme morning and evening/extreme evening types were selected (i.e. intermediate types were excluded) to maximise the likelihood of detecting an association, if any, between the *PER3* ^G^ and *PER3* ^C^ variants and diurnal preference. All required data were available for n = 103 morning/extreme morning types and n = 106 evening/extreme evening types. This population was also shown to be in Hardy-Weinberg equilibrium (χ^2^ = 0.248, DF = 1, p = 0.618), with no significant differences in SNP genotype distribution between females and males (χ^2^ = 0.184, DF = 2, p = 0.912). The distribution of the combined genotypes of both polymorphic sites is shown in Table 2, for morning and evening types separately. Analysis of this sample confirmed the absence of any preferential association between the VNTR polymorphism and diurnal preference (Cochran-Armitage test χ^2^ = 0.467, DF = 1, p = 0.494). By contrast, for the SNP rs228697 an association was observed between the *PER3* ^G^ variant and morningness (Cochran-Armitage test χ^2^ = 6.998, DF = 1, p = 0.008, OR = 2.10, 95% CI = 1.21–3.65). Allelic co-occurrence at the two sites (Table 2) shows that the *PER3* ^G^ variant was almost in complete linkage disequilibrium with the *PER3* ^4^ variant (D′ = 0.912). The conditional haplotype test showed a significant association of the *PER3* ^G^-*PER3* ^4^ haplotype with morningness (likelihood ratio χ^2^ = 8.240, DF = 2, p = 0.016, OR = 2.19, 95% CI = 1.17–4.08). This was mostly due to the *PER3* ^G^ association with morningness. Both allelic and haplotypic associations remained significant (p < 0.05) after correction for multiple comparisons.

**Table 2:** Genotypes and minor allele frequencies at the two *PER3* polymorphic sites, by diurnal preference.

rs228697	rs57875989	Morning (n = 103)	MAF(G)	MAF(5)	Evening (n = 106)	MAF(G)	MAF(5)	Total (n = 209)	MAF(G)	MAF(5)
G/G	4/4	0.058	0.204	0.340	0.000	0.108	0.373	0.029	0.155	0.356
C/G	4/4	0.184			0.085			0.134		
C/C	4/4	0.223			0.302			0.263		
G/G	4/5	0.000			0.000			0.000		
C/G	4/5	0.107			0.123			0.115		
C/C	4/5	0.282			0.358			0.320		
G/G	5/5	0.000			0.000			0.000		
C/G	5/5	0.000			0.009			0.005		
C/C	5/5	0.146			0.123			0.134		

MAF: Minor Allele Frequency. G, C and 4, 5 refer to the alleles at rs228697 and rs57875989, respectively. The *PER3* ^G^ allele (rs228697) was significantly more represented in morning types (OR = 2.10, p = 0.008). Genotypes at the two sites showed an almost complete linkage disequilibrium between the *PER3* ^G^ and *PER3* ^4^ (rs57875989) alleles (D′ = 0.912). This *PER3* ^G^ - *PER3* ^4^ haplotype also showed a significant association with morningness (OR = 2.19, p = 0.016).

Individuals with one or two *PER3* ^G^ alleles woke up significantly earlier, tended to sleep less and reported significantly worse sleep quality compared to their *PER3* ^C^ */PER3* ^C^ counterparts (diaries available for 117 subjects; Table 3). However, these differences were not significant after correction for multiple comparisons (FDR adjusted p > 0.05). Although sample numbers became small, individuals with two *PER3* ^G^ alleles (n = 5) tended to exhibit even earlier sleep-wake times than their counterparts with one or no *PER3* ^G^ alleles [i.e. wake up time (decimal h): 6.8 ± 0.8 (two *PER3* ^G^ alleles) vs. 7.7 ± 1.5 (one *PER3* ^G^ allele) vs. 8.2 ± 1.2 (no *PER3* ^G^ alleles), p = 0.057]. Individuals with any degree of *PER3* ^G^ - *PER3* ^4^ association (i.e. heterozygotes or homozygotes for the *PER3* ^G^ together with one or two *PER3* ^4^) woke up significantly earlier, slept less and reported worse sleep quality compared to their counterparts without the association (Table 3). However, these differences were not significant after correction for multiple comparisons (FDR adjusted p > 0.05).

**Table 3:** Sleep-wake timing and sleep quality indices in relation to the presence/absence of the *PER3* ^G^ allele and the *PER3* ^G^ - *PER3* ^4^ association.

	*PER3* ^G^ Present (n = 33)	*PER3* ^G^ Absent (n = 84)	*PER3* ^G^ - *PER3* ^4^ Present (n = 32)	*PER3* ^G^ - *PER3* ^4^ Absent (n = 85)
Bed time (decimal h)	24.0 ± 1.5	24.3 ± 1.3	24.1 ± 1.5	24.3 ± 1.3
Time to fall asleep (min)	16.0 ± 11.9	13.2 ± 10.5	16.4 ± 12.0	13.1 ± 10.5
Sleep onset (decimal h)	24.6 ± 1.6	24.9 ± 1.3	24.7 ± 1.5	24.8 ± 1.4
Awakenings (n)	0.9 ± 1.0	0.8 ± 0.7	0.9 ± 1.1	0.8 ± 0.7
Wake up time (decimal h)	7.6 ± 1.4*	8.2 ± 1.2	7.6 ± 1.4 *	8.1 ± 1.2
Get up time (decimal h)	8.0 ± 1.4	8.5 ± 1.2	8.0 ± 1.4	8.5 ± 1.2
Naps (n)	0.1 ± 0.2	0.2 ± 0.3	0.1 ± 0.2	0.2 ± 0.2
Length of sleep (h)	6.9 ± 0.9	7.3 ± 0.8	6.9 ± 0.8*	7.3 ± 0.8
Sleep efficiency (%)	87.5 ± 8.7	89.2 ± 7.0	87.3 ± 8.8	89.3 ± 7.0
Sleep quality (1–10 scale)	4.2 ± 1.5*	3.7 ± 1.3	4.2 ± 1.5*	3.7 ± 1.3
PSQI global score (0–21)°	6.9 ± 4.2*	5.4 ± 2.9	7.1 ± 4.2*	5.4 ± 2.9

*p < 0.05 on direct comparisons, not confirmed after correction for multiple comparisons (FDR adjusted p > 0.05). °Data available for 37/90 participants with/without the *PER3* ^G^ allele and for 36/91 participants with/without the *PER3* ^G^ - *PER3* ^4^ association. PSQI: Pittsburgh Sleep Quality Index.

**Figure 1:**
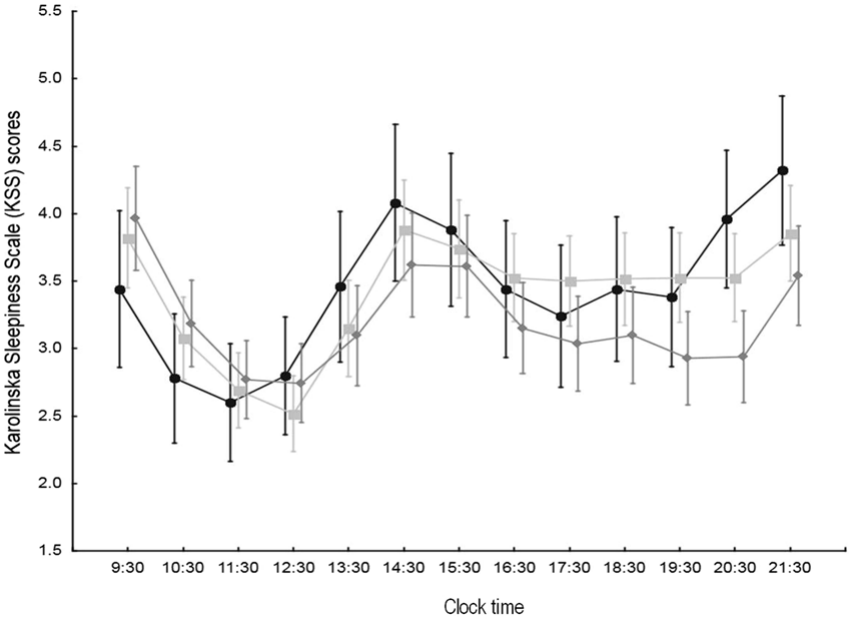
Karolinska Sleepiness Scale scores (1–9, with 9 = maximum sleepiness; mean ± 95% CI) over the waking hours of the day in *PER3* ^5^ */PER3* ^5^ (black circles, n = 59), *PER3* ^4^ */PER3* ^5^ (light grey squares, n = 137) and *PER3* ^4^ */PER3* ^4^ (dark grey diamonds, n = 135) subjects. Effect of genotype: p = 0.195, time: p < 0.001, genotype*time: p = 0.029.

**Figure 2:**
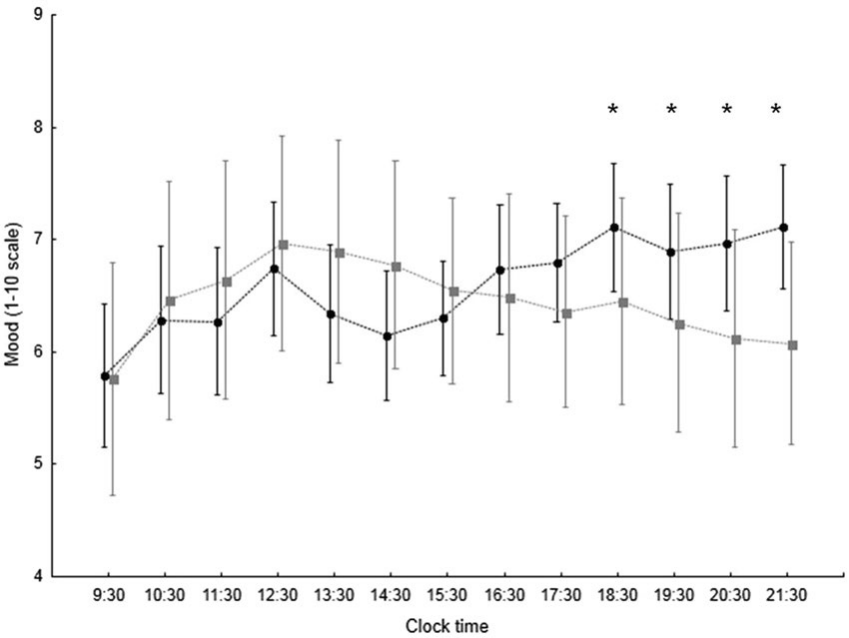
Mood scores (1–10 scale, 10 = best; mean ± 95% CI) over the waking hours of the day in individuals with (grey squares, n = 19) and without (black circles, n = 53) the *PER3* ^G^ - *PER3* ^4^ association. Effect of genotype: p = 0.756, time: p = 0.045, genotype*time: p = 0.020. *Post hoc significant with p < 0.05 at 19:30 h and 20:30 h, p < 0.01 at 18.30 h and 21:30 h.

The KSS and mood scales were completed by 388 subjects (36%, 278 females). Sleepiness showed significant variation over the waking hours of the day, and there was a significant interaction genotype*time for the VNTR polymorphism (n = 331). In particular, *PER3* ^5^ */PER3* ^5^ subjects (n = 59) were less sleepy in the morning and more sleepy in the early afternoon/early evening compared to their *PER3* ^4^ */PER3* ^4^ (n = 135) and *PER3* ^4^ */PER3* ^5^ (n = 137) counterparts (genotype: p = 0.195, time: p < 0.001, genotype*time: p = 0.029; *post hoc* significant at several time points; Fig. 1). There was no significant relationship between KSS scores and the SNP alone or in combination with the VNTR polymorphism (n = 72). Self-rated mood also showed significant variation over the waking hours of the day but no significant association with either polymorphism alone (n = 331 for the VNTR polymorphism and n = 72 for the SNP, respectively). However, when the *PER3* ^G^ - *PER3* ^4^ association was considered, there was a significant interaction between genotype and mood, individuals with the association (n = 19) having a significantly worse mood in the early evening hours compared to those without (n = 53) (genotype: p = 0.756, time: p = 0.045, genotype*time: p = 0.020; *post hoc* significant from 18:30 to 21:30 h; Fig. 2).

### Response to light

**Figure 3:**
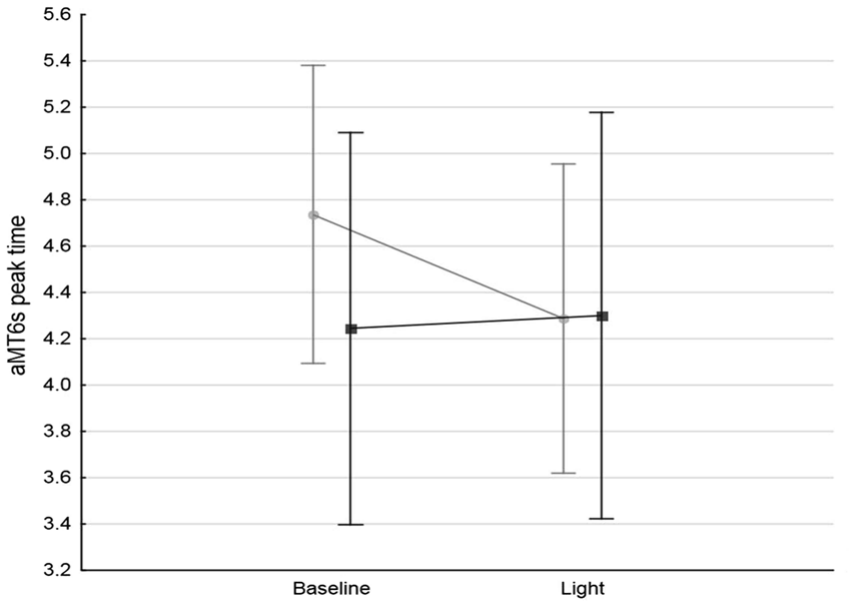
6-sulfatoxymelatonin (aMT6s) acrophase in the *PER3* ^4^ */PER3* ^4^ (light grey, n = 19) and *PER3* ^5^ */PER3* ^5^ (dark grey, n = 11) subjects, before and after light administration. Effect of genotype: p = 0.646, time: p = 0.101, genotype*time: p = 0.037.

No significant differences were observed in sleep timing and/or aMT6s rhythms between *PER3* ^4^ */PER3* ^4^ and *PER3* ^5^ */PER3* ^5^ individuals at baseline (Table 4). After light administration, 15 subjects (12 *PER3* ^4^ */PER3* ^4^, 3 *PER3* ^5^ */PER3* ^5^) showed an advance in their aMT6s acrophase, 3 a delay in aMT6s acrophase (all *PER3* ^5^ */PER3* ^5^) and 12 no significant change (7 *PER3* ^4^ */PER3* ^4^, 5 *PER3* ^5^ */PER3* ^5^). Thus, when the direction of phase shift was accounted for, significant differences were observed between the response to light in the two genotypes, with *PER3* ^4^ */PER3* ^4^ subjects advancing their aMT6s acrophase by approximately half an hour compared to their *PER3* ^5^ */PER3* ^5^ counterparts (−26 ± 32 vs. +3 ± 43 min; p = 0.037; Fig. 3). Absolute value changes (i.e. not taking into account of their direction) were comparable in the two groups (30 ± 29 min in *PER3* ^4^ */PER3* ^4^ participants vs. 30 ± 30 min in *PER3* ^5^ */PER3* ^5^ participants). No significant changes were observed in other aspects of the aMT6s rhythms (mesor, amplitude, total amount produced) in relation to light administration.

**Table 4:** Baseline sleep diary and urinary aMT6s rhythm indices, by genotype.

	Sleep diary variables			Urinary aMT6s rhythm cosinor indices		
	Sleep onset (clock time, h:min)	Wake-up (clock time, h:min)	Awakenings (n)	Mesor (pg/ml)	Amplitude (pg/ml)	Achrophase (clock time, h:min)
*PER3* ^4^ */PER*3^4^ (n = 19)	00:49 ± 01:12 (23:03–03:52)	07:39 ± 01:16 (05:46–10:39)	1.0 ± 0.9 (0.0–4.3)	13.91 ± 6.55 (3.52–26.47)	18.76 ± 8.85 (5.37–35.84)	04:45 ± 01:31 (02:12–07:11)
*PER3* ^5^ */PER3* ^5^ (n = 11)	00:33 ± 01:06 (22:55–02:25)	07:49 ± 00:51 (06:29–08:54)	1.2 ± 0.8 (0.3–2.3)	12.10 ± 6.92 (4.47–28.67)	15.68 ± 10.28 (4.00–39.39)	04:14 ± 01:02 (02:04–05:30)

Data are expressed as mean ± SD (range). aMT6s: 6-sulphatoxymelatonin.

*PER3* ^4^ */PER3* ^4^ subjects (n = 19) also exhibited a trend (0.05 < p < 0.1) for a stronger response to light in terms of an earlier sleep onset and wake up times after light administration compared to their *PER3* ^5^ */PER3* ^5^ counterparts (n = 11). In both *PER3* ^4^ */PER3* ^4^ and *PER3* ^5^ */PER3* ^5^ groups, the course of light treatment significantly reduced the number of awakenings (from approximately 1 per night to 0.5 per night; genotype: p = 0.194, time: p = 0.003, genotype*time: p = 0.694).

While the SNP was only available in 12 individuals who underwent light administration and statistical power was insufficient for a full analysis, the tendency for individuals with any degree of *PER3* ^G^ - *PER3* ^4^ association (one or two *PER3* ^G^ together with one or two *PER3* ^4^, n = 3 out of the 12 available) to show earlier wake-up times and an earlier aMT6s acrophase was confirmed.

## Discussion

This is the first study carried out in a large, healthy population of Caucasian individuals living in Northern Italy regarding the relationship of two clock gene *PER3* polymorphic sites (the rs228697 SNP and the *PER3* ^4^-*PER3* ^5^ rs57875989 VNTR polymorphism) and a set of sleep-wake phenotypes, including diurnal preference, sleep-wake timing and mood.

While several studies have implicated the *Period* genes *PER1* and *PER2* in the molecular timekeeping of the master circadian clock, the role of *PER3* is less clear. In a study assessing *PER1*-promoter-driven luciferase expression in cultured suprachiasmatic nuclei (SCN), pituitary, and lung explants from *PER2* ^−/−^ and *PER3* ^−/−^ mice, Pendergast *et al*.^13^ observed that the period of *PER1*-*luc* expression in *PER2* ^−/−^ SCN explants was 1.5 hours shorter than that of *PER2* ^+/+^. In the same study, *PER3* ^−/−^ and *PER3* ^+/+^ mice exhibited comparable SCN explants, while the period/phase of their pituitary and lung explants were different, and were abnormal in *PER3* ^−/−^. These observations suggest that the function of each *PER* gene may be tissue-specific. In a subsequent study, Pendergast *et al*.^14^ observed shortening of periods in some peripheral tissues but not in the SCN of *PER3* ^−/−^ mice compared to wild-types. Such period shortening resulted in advanced phases, indicating that some degree of internal misalignment may occur in *PER3* ^−/−^ mice. Based on the circadian features/light sensitivity of both *PER3* ^−/−^ mice and rare *PER3* polymorphic variants, Zhang *et al*.^15^ have recently hypothesised that PER3 may affect the clock via a stabilising function on PER1/PER2.

In the present study, no relationship was observed between the *PER3* ^4^ – *PER3* ^5^ VNTR polymorphism and diurnal preference. The available literature is conflicting in this respect, with at least three studies documenting this association^7, 16, 17^ and others failing to replicate it^18–20^. Several factors could explain these discrepancies. For example, Pereira and colleagues^16^ have suggested that latitude may be implicated, together with differences in the length of days, climate and temperature variations throughout the year. However, two of the conflicting studies were based in the United Kingdom, thus on populations sharing similar environmental conditions^7, 18^. It is reasonable that the diurnal preference phenotype might be multifactorial in origin, with genetic factors being more or less obvious depending on the features of the population, and the varying weight of other factors.

Melatonin production, subjective sleepiness and waking slow electroencephalographic activity are reduced by the exposure to 460 nm blue monochromatic light during the biological night^21, 22^. Such light responses have been reported to be stronger in *PER3* ^5^ */PER3* ^5^ compared to *PER3* ^4^ */PER3* ^4^ healthy subjects^8^, thus it has been suggested that *PER3* variants may contribute to the inter-individual variability of the non visual responses to light^8^. All protocols testing such a hypothesis have been performed within the context of light-induced suppression of nocturnal melatonin production^8, 21^, with administration of light at night. In the clinical/therapeutic setting, however, bright light is often used to advance circadian phase, and is thus delivered at the beginning of the day^23^. Hence our attempt to test light responses in relation to genotype in the setting of morning light administration. In our study, despite large within group variability, morning light showed more prominent phase shifting effects in *PER3* ^4^ */PER3* ^4^ compared to *PER3* ^5^ */PER3* ^5^ subjects. This is reasonable, as supplementation of morning light may be more effective in individuals who are intrinsically less sensitive to light (*PER3* ^4^ */PER3* ^4^ subjects^8^) and thus less likely to have already benefited from the alerting effect of getting up and being exposed to standard room lighting in the early morning. This interpretation is supported by our data on the time-course of subjective sleepiness in *PER3* ^5^ */PER3* ^5^ versus *PER3* ^4^ */PER3* ^4^ subjects. Interestingly, all three subjects who showed a somewhat paradoxical response to morning light, delaying their aMT6s acrophase, were *PER3* ^5^ */PER3* ^5^, in line with previous findings on light sensitivity in this group^8^. Morning light supplementation reduced the number of night awakenings in all treated subjects, confirming its sleep consolidating effects^9^. These data are clinically relevant as they could help: *i*) understanding blunted and excessive/paradoxical responses to light administration; *ii*) refining patient selection and monitoring during a course of chronotherapy.

In this study, we observed a novel, strong association between the *PER3* ^G^ allele (rs228697 SNP) and morningness, and an even stronger one between the *PER3* ^G^ - *PER3* ^4^ haplotype and morningness. This was confirmed by daily sleep diaries, which documented earlier sleep onset (trend) in *PER3* ^G^ and *PER3* ^G^ - *PER3* ^4^ subjects. However, these differences were not significant after correction for multiple comparisons. Our findings contrast, to some extent, with those of Hida and colleagues^11^, who documented a weak albeit significant association between the *PER3* ^G^ allele and eveningness. Interestingly, and despite large within group variability, in our study *PER3* ^G^ - *PER3* ^4^ subjects also showed a different time-course of their mood over the waking hours, with significantly lower mood scores in the late afternoon/early evening. Rare *PER3* variants have previously been linked to depressive behaviour both in humans with advanced sleep phase syndrome and in a mouse model mimicking rare *PER3* human mutations^15^. In addition, progressive worsening of mood has been documented over the waking hours of the day in humans^24^. This may of course be modulated by diurnal preference.

Haplotype frequency pattern analysis (Table 2) suggests a tentative parsimonious phylogeny relationship accounting for the origin of the four haplotypes. We hypothesize that *PER3* ^G^ - *PER3* ^4^ and *PER3* ^C^ - *PER3* ^4^ haplotypes could be the ancestral ones, and that the 4-units to 5-units expansion of the repeated region occurred on a *PER3* ^C^ - *PER3* ^4^ background, thus originating the *PER3* ^C^ - *PER3* ^5^ haplotype. A very low recombination rate between the *PER3* ^C^ - *PER3* ^5^ and *PER3* ^G^ - *PER3* ^4^ haplotypes, due to the very small distance between the two polymorphic sites (2348 base pairs), could thus explain the almost complete absence of the *PER3* ^G^ - *PER3* ^5^ haplotype. Interestingly, the carrier and estimated haplotype frequencies for the rs228697 and rs57875989 polymorphisms described by Ebisawa and colleagues^6^ reflect the distribution observed in this study, with no *PER3* ^*G*^ *- PER3* ^5^ association.

The association between the *PER3* ^G^ - *PER3* ^4^ haplotype and morningness could reflect the specific effects of the PER3^A^ variant on the circadian molecular cycle. As pointed out by Hida and colleagues^11^, the P864A amino acid substitution affects the hydropathy index of the protein, altering its secondary structure and its phosphorylation dynamics. Indeed, the substitution could disrupt two potential SH3-binding domains [(LPDPP-864) and (PP-864-VCP)] which could, in turn, impinge on the interaction between PER3 and NCK. NCK is a scaffolding protein with SH3 domains which has been implicated in the PER3 interaction with a CKI. Such interaction regulates PER accumulation/stability throughout the circadian cycle^12^. In this scenario, it can be hypothesised that the PER3^A^ protein is more stable and accumulates faster in the cytoplasm compared to the PER3^P^. Therefore, PER3^A^ is likely to enter the nucleus earlier than PER3^P^ to exert a negative feedback on the transcription of *PER* and *CRY* genes. This would result in shorter periodicity in free running conditions, and phase advancing of some circadian phenotypes, which fits with our observed association between the ‘faster accumulating’ PER3^A^ protein and morningness. While PER3 appears to affect mainly peripheral clocks^13, 14^, it cannot be excluded that at least some of these effects may feedback to the SCN, contributing to the modulation of the phase of sleep timing. Based on experiments in primary dermal cells, Brown *et al*.^2^ suggested that human diurnal preference may be influenced by cellular components that affect the amplitude and phase of the circadian oscillator. Ferrante *et al*.^25^ observed that *PER3* circadian expression in a peripheral tissue (hair follicle) of morning types was 1.5 hours earlier than that of intermediate types, and 3 hours earlier than that of evening-types. If our hypothesis of the effects of the P864A polymorphism on the molecular mechanisms underlying the oscillation is correct, a 2–3 hour phase difference in peripheral clocks in *PER3* ^C^ and *PER3* ^G^ bearing individuals could modulate the phase of their sleep.

The contribution of the *PER3* ^4^ VNTR variant, albeit modest, in association with *PER3* ^G^ in defining a morning phenotype is also in line with the proposed model, because the number of putative phosphorylation sites situated on the repeats are fewer in *PER3* ^4^ compared to *PER3* ^5^. This could result in faster cytoplasm accumulation kinetics and, in turn, in negative feedback and phase advancing. It is possible that this may also affect the sensitivity to light. Indeed, it has been demonstrated that light induces a larger phase delay (or smaller phase advance) in different, rare human *PER3* variants, which results in abnormal entrainment^15^.

Of the five non synonymous SNPs which Ebisawa and colleagues originally implicated in the pathogenesis of delayed sleep phase syndrome^6^, one (rs10462020, which causes a V674G substitution) has also been associated with diurnal preference by Johansson and colleagues^26^. Another one (rs2640909) causes a M1037T substitution, which alters the hydrophobicity of the PER3 protein, possibly affecting PER3 auto-feedback^27^. This polymorphism was associated with morningness by Ojeda and colleagues^27^ but not by Hida and colleagues^11^.

In conclusion, significant associations were observed between the *PER3* ^G^ polymorphic variant in rs228697 (alone and in combination with the *PER3* ^4^ variant in rs57875989) and morningness, as well as the course of mood over the waking hours of the day.

## Methods

### Participants

A sample of 786 subjects describing themselves as Caucasian and living in Northern Italy [531 females, age 28.6 ± 12.5 (range 18–79)] were recruited in Padova, Italy, during popular science events (January 2012, September 2013 and September 2014). Subjects were excluded if they had significant medical history, any chronic disease, or took regular medication, except for oral contraceptives. All volunteers were asked to donate a buccal DNA sample (brushing the internal cheek surface firmly for about 15 seconds with a swab; n = 679 accepted), and to complete a sleep-wake assessment including diurnal preference [Horne - Östberg questionnaire (HÖ questionnaire)^28^], night sleep quality [Pittsburgh Sleep Quality Index (PSQI)^29^], Karolinska Sleepiness Scale (KSS)^30^], mood (visual-analogue 1–10 scale) and habitual sleep timing (sleep diaries). The HÖ questionnaire was completed during the popular science initiative, with the same researchers providing instructions and answering any questions. The remaining questionnaires and diaries were completed by the participants at home, in the subsequent two-three weeks. Addressed, stamped envelopes were provided; 550 (51%) were returned, with partial or complete information. Participants who provided a readable, viable e-mail address (98%) were subsequently sent a summary report of their sleep-wake assessment and genotype. A brief description of the questionnaires and diaries utilized is provided below.

Study protocols were approved by the Padova University Hospital Ethics Committee (Medical Bio-bank and 3639/AO/15 for light administration) and conducted according to the Declaration of Helsinki (Hong Kong amendment) and good clinical practice (European) guidelines. All methods were performed in accordance with the above ethical guidelines and regulations. All participants provided written, informed consent.

### Sleep-wake assessment

The HÖ questionnaire was used to assess diurnal preference. The total score ranges from 16 to 86: scores <41 define evening (extremely evening <30) types, scores ≥59 define morning (extremely morning ≥70) types, and scores between 42 and 58 intermediate types^28^.

The PSQI was used to assess night sleep quality over the preceding month. Questionnaire responses generate seven components (subjective sleep quality, sleep latency, sleep duration, habitual sleep efficiency, sleep disturbances, use of sleeping medication, and daytime dysfunction), which are summated to provide the PSQI global score (range: 0–21); scores of >5 identify ‘poor’ sleepers^29, 31^.

The KSS was used to assess daytime sleepiness. Participants rated their subjective sleepiness over the previous 10 minutes, from 1 (very alert) to 9 (very sleepy, fighting sleep, difficulty staying awake)^30^. In this particular instance, participants were provided with a set of KSS sheets and asked to complete them on an hourly basis over the waking hours of a typical working/studying day, from get-up to bed-time.

Mood visual-analogue scale sheets were also provided (“Please rate your mood from 1–10 on this scale”, with 10 being marked as best) and participants asked to complete them on an hourly basis over the waking hours of a typical working/studying day, from get-up to bed-time^32^.

Sleep diaries were completed daily that included a record of bed-time, sleep onset, time to fall asleep, wake-up time, get-up time, number/duration of any night awakenings or daytime naps. Relevant metrics were calculated, including sleep onset latency, wakefulness after initial sleep onset, total sleep time, total time spent in bed, sleep efficiency (time asleep out of total time in bed, as a percentage), and a subjective appraisal of each night’s sleep quality (1–10, with 10 being the negative extreme)^33^. Participants were instructed to complete the sleep diary each morning, after every night sleep, for 12 days, to include a week-end. Sleep diaries were also completed during the light administration protocol (*vide infra*) and sleep data were considered both singly and as averages pre- (study days 2–4, Tuesday to Thursday) and during light administration (study days 9–11, also Tuesday to Thursday).

### Genotyping

#### *PER3*^4^ – *PER3*^5^ VNTR (Genoma, Build GRCh38, chrom1, start 7829867)

Genotyping was performed using polymerase chain reaction (PCR) with the primers described by Ebisawa *et al*.^6^ (Supplementary Table 1). The polymerase mixture was prepared in a 20 µl final volume and was composed of 10.6 μl sterile water; 4 µl 5X Taq buffer; 1 μl of 10 mM dNTPs mix; 1 μl of 10 µM Reverse primer; 1 μl of 10 µM Forward primer; 2 μl Genomic DNA; and 2U of GoTaq DNA Polymerase (Promega). The amplification conditions were: 94 °C for 3 minutes, then 30 cycles of 94 °C for 1 minute, 55 °C for 1 minute, 72 °C for 1 minute and a final elongation of 72 °C for 10 minutes. Agarose gel electrophoresis was used to identify whether individuals were heterozygous or homozygous for either of the *PER3* repeat alleles. For the subgroup of 209 subjects who were also genotyped for SNP rs228697 (*vide infra*), genotyping by gel electrophoresis was validated by GeneScan analysis by using the same primer pair proposed by Ebisawa *et al*.^6^ but with a HEX-labelled version of the forward primer. Detailed primer sequences are listed in Supplementary Table 1. A negative and two positive controls (*PER3* ^4^ and *PER3* ^5^) were included in each test performed.

#### SNP rs228697 (C/G− > P/A) (Genoma, Build GRCh38, chrom1, 7827519)

The detection of the C2590G (P864A) polymorphism was performed by a double fluorescent tetra-primer amplification refractory mutation system PCR (dF-T-ARMS-PCR)^34^. Two allele-specific primer pairs were designed according to the requirements of the df-T-ARMS-PCR protocol. The specificity of each allele-specific primer is conferred by the identity of the terminal 3′ nucleotide with one of the two possible single nucleotide variations. The two different fluorescent dyes (FAM and HEX) used to specifically label the two allele-specific primer pairs, as well as the significantly different length of their amplicons, allowed for easily distinguishable signals by GeneScan analysis (Peak Scanner Software v1.0; Thermo Fisher Scientific). Primer sequences are detailed in Supplementary Table 1. PCR was performed in a 50 µl final volume and was composed of 27.6 μl sterile water; 10 µl 5X Taq Buffer; 1 μl of 10 mM dNTPs mix; 1 μl of 10 µM Forward and Reverse primers; 5 μl Genomic DNA; and 2U GoTaq DNA Polymerase (Promega). The thermal cycling conditions were as follows: 2 minutes denaturation at 95 °C, followed by 35 cycles of 15 seconds denaturation at 95 °C, 20 seconds annealing at 57 °C, 1 minute elongation at 72 °C, and a final 3-min elongation cycle at 72 °C. Capillary electrophoresis was performed with an ABI 3100 Genetic Analyzer (Applied Biosystems) at BMR Genomics (Padova, Italy). The size of the products was determined using the GeneScan 400HD ROX size standard (Thermo Fisher). A negative and two positive controls (*PER3* ^C^ and *PER3* ^G^) were included in each test performed.

### Morning light administration protocol

A subgroup of 30 subjects who were homozygous for the length polymorphism [19 *PER3* ^4^ */PER3* ^4^ (27 ± 9 yrs, 15 females) and 11 *PER3* ^5^ */PER3* ^5^ (26 ± 10 yrs, 8 females)] underwent a course of morning light administration, whilst monitoring sleep-wake patterns and melatonin rhythmicity.

#### Experimental design

The study was conducted over 12 days and sleep-wake diaries completed daily (Table 5). The KSS was completed hourly over the waking hours of days 1 (pre-light) and 11 (end of light) (Table 5). Two separate 56-hour urine collections (over 2 days and 3 nights, at 4 hour intervals during the day and 8 hour intervals at night) were obtained on study days 1–3 (pre-light) and 9–11 (end of light) (Table 5) for assessment of the urinary melatonin metabolite 6-sulfatoxymelatonin (aMT6s; *vide infra*) rhythms. Participants were provided with a Light Box (SAD3 10000 lux - E.M.S. srl Bologna, Italy) and instructed to expose themselves to light daily for 45 minutes, immediately after getting-up. They were asked to place the Light Box at approximately 60 cm from their head, to one side, and to direct their gaze at the Light Box every 10–15 minutes. If they moved away from the box (for example to go to the toilet or get dressed) they were instructed to make up the lost time at the end of the session. During the entire study period, subjects were asked to maintain regular sleep-wake schedules and avoid significant changes to their habitual consumption of coffee, tea, chocolate and alcoholic beverages. They were also asked to avoid exposure to very bright light and use of mobiles/tablets in the evening and at night. Compliance was checked by phone every third day by one of the researchers.

**Table 5:** Light administration study design.

Study Day											
1 Mon	2 Tue	3 Wed	4 Thu	5 Fri	6 Sat	7 Sun	8 Mon	9 Tue	10 Wed	11 Thu	12 Fri
Baseline questionnaires plus provision of Light Box	Hourly KSS										Hourly KSS
				Morning light administration							
	Urine collection for aMT6s								Urine collection for aMT6s		
Sleep diaries											

aMT6s: 6-sulphatoxymelatonin. KSS: Karolinska Sleepiness Scale.

#### 6-sulfatoxymelatonin (aMT6s) assessment

Urinary samples were kept at −20 °C and aMT6s concentrations measured by radioimmunoassay (Stockgrand Ltd.). Urine samples were analyzed in duplicate and all samples from the same subject were included in the same assay. The inter-assay coefficients of variation (CVs) were 13.3% at 2.4 ng/mL, 8.9% at 10.4 ng/mL, and 15.4% at 17.5 ng/mL (n = 16 at each concentration).

The aMT6s rhythms were evaluated using cosinor analysis, which is based on the least square approximation of the time series using a cosine function with a period of 24 hours^35^. The following parameters were obtained: acrophase time (time of peak aMT6s concentration, or maximum of the fitted cosinor function), mesor (mean aMT6s value for all the samples included in the cosinor analysis) and amplitude (difference between the mesor and the peak aMT6s concentrations). Data were considered acceptable if the cosinor fit, or the likelihood of the data points fitting a cosine curve as opposed to a straight line, was significant at the 95% level (p < 0.05)^36^. All parameters were assessed both pre- and post-light administration and the difference in aMT6s acrophase (peak) time between the pre- and post-light administration was also computed and considered significant when >15 min.

### Statistical Analysis

Data are presented as mean ± standard deviation (SD) or 95% Confidence Interval (CI). Clock times are presented as decimal hours (h) within the results section, and as h:mins in Tables and Figures, to facilitate reading, where possible.

Hardy-Weinberg equilibrium was tested by the Pearson χ^2^. Association testing between single polymorphisms and diurnal preference was performed using the Cochran-Armitage test (PLINK open-source whole genome association analysis toolset; http://pngu.mgh.harvard.edu/purcell/plink/)^37^. Haplotypic testing was conducted using the conditional haplotype-based test module, also on PLINK, which estimates haplotypes to be tested from unphased genotypes. Experiment-wide correction for multiple testing was applied (FDR method, n = 3 tests).

The Student t/Mann-Whitney U test (correction for multiple testing applied where appropriate) and ANOVA/Kruskal-Wallis ANOVA were used to compare sleep-wake variables between two and multiple groups, as appropriate (post-hoc: Tukey/multiple comparisons of mean ranks). Repeated measures ANOVA was utilized to assess the time-course of variables and/or their changes in relation to light administration (post-hoc: Tukey test) (Statistica 13.1, Dell. Round Rock, Texas, US). As hourly KSS/mood data were available for most subjects in the time slot between 9:30 and 21:30 h (at earlier/later times, the numbers decreased significantly), this was the time interval utilized for analysis.

## Electronic supplementary material

Supplementary Table 1
