# Supplementary material for: Diurnal preference, mood and the response to morning light in relation to polymorphisms in the human clock gene PER3
Source: Sci Rep. 2017 Jul 31;7:6967. doi: 10.1038/s41598-017-06769-w (PMC5537342; doi:10.1038/s41598-017-06769-w)
Supplement: Supplementary file 1 — Supplementary Table 1 [file 41598_2017_6769_MOESM1_ESM.doc]

Diurnal preference, mood and the response to morning light in relation to polymorphisms in the human clock gene *PER3*

Turco M,^1^ Biscontin A,^2^ Corrias M, ^1,3^ Caccin L,^2^ Bano M,^1^ Chiaromanni F,^1^ Salamanca M,^1^ Mattei D,^1^ Salvoro C,^2^ Mazzotta G,^2^ De Pittà C,^2^ Middleton B,^3^ Skene DJ,^3^ Montagnese S,^1*^ Costa R ^3*^

*^1^Department of Medicine, University of Padova, Padova, Italy*

*^2^Department of Biology, University of Padova, Padova, Italy*

*^3^Chronobiology, Faculty of Health and Medical Sciences, University of Surrey, Guildford, United Kingdom*

**Co-senior author*

**Supplementary Table 1.** Primer specifics for the multiplex GeneScan analysis

| **Polymorphism** | **Primer** | **Sequence** | **Fluorescent dye (5’ terminus)** | **Amplicon length (bp)** |
| --- | --- | --- | --- | --- |
| rs57875989 | VNTR_Forward | CAGTGTGTTACAGGCAACAATGGCAG | HEX | 328 (H5)  274 (H4) |
|  | VNTR_Reverse | CTGATGCTGCTGAACCAGTTCTGG |  |  |
| rs228697 | C_Forward | CTCACCCGGAAGAGAATACG |  | 163 |
|  | C_Reverse | CGACAACAGAGGACAGACAGG | HEX |  |
|  | G_Forward | GTTTTCCTGCCTGACCCCG | FAM | 179 |
|  | G_Reverse | GCCTCCCACTTTTCCTCCTC |  |  |
